# CHILDHOOD CUTANEOUS VASCULITIS: A COMPREHENSIVE APPRAISAL

**DOI:** 10.4103/0019-5154.53179

**Published:** 2009

**Authors:** Aparna Palit, Arun C Inamadar

**Affiliations:** *From the Departments of Dermatology, Venereology and Leprosy, BLDEA's SBMP Medical College, Hospital and Research Center, Bijapur, Karnataka, India*

**Keywords:** *Behçet's disease*, *cutaneous polyarteritis nodosa*, *cutaneous vasculitis*, *Henoch-Schönlein purpura*, *Kawasaki disease*

## Abstract

Cutaneous vasculitides in childhood are rare and often present with clinical features distinct from adults. Diagnosis of cutaneous vasculitides in children was difficult because of lack of a satisfactory classification systems for this age group. A new international classification system for childhood vasculitis has been discussed in the following section along with important clinical features, diagnostic modalities, and recent therapeutic developments of important vasculitides in children.

## Introduction

Vasculitis is rare in the pediatric age group and differs from the adult disease in various aspects. The two most commonly encountered vasculitides in children are Henoch-Schönlein purpura and Kawasaki disease. These disorders in children may differ from adults in clinical spectrum, disease severity, and prognosis.

Some vasculitic disorders present with predominant cutaneous manifestations grouped commonly as cutaneous vasculitis, though visceral involvement may be part of the disease. Others are multi-organ vasculitides where skin is involved as a part of the disorder. Cutaneous vasculitis may also be part of other systemic diseases like collagen vascular disorders, infections, and may follow administration of exogenous agents like drugs. Since cutaneous vasculature is one of the most easily appreciable in the human body, vasculitic disorders manifest readily on the skin.

## Classification

Since its recognition, there was no separate classification system for pediatric and adult vasculitides. None of the available classification systems (American College of Rheumatology [ACR], Chapel Hill Consensus Conference [CHCC]) address the pediatric issues adequately. However, childhood vasculitides need special attention and recognition.

To overcome these shortcomings, a new classification system for pediatric vasculitides has been proposed (International Consensus Conference, Vienna, June 2005), which has been presented in Table 1.[1] The basis for this classification system is vessel size (groups I, II, and III) and Group IV includes entities difficult to categorize.[1] At the same time, a new set of classification criteria and definitions for important childhood vasculitides has been proposed.[1] This system has been mainly developed for use by the pediatricians and still awaits appropriate validation using patient and control groups.[1] However, it appears to address the issues related to pediatric cutaneous and systemic vasculitides adequately and may be useful for the dermatologists as well.

**Table 1:** Proposed classification system for childhood vasculitis[1]

Predominantly large vessel vasculitis
Takayasu's arteritis
Predominantly medium-sized vessel vasculitis
Childhood polyarteritis nodosa Cutaneous polyarteritis nodosa Kawasaki disease
Predominantly small-vessel vasculitis
A. Granulomatous Wegener's granulomatosis Churg-Strauss syndrome B. Non granulomatous Microscopic polyangiitis Henoch-Schönlein purpura Isolated cutaneous leucocytoclastic vasculitis Hypocomplementemic urticarial vasculitis
Other vasculitides
Behçet's disease Vasculitis secondary to infections, malignancy, drugs Isolated vasculitis of CNS Cogan's syndrome Unclassified

The following discussion will focus on cutaneous vasculitis and cutaneous manifestations of systemic vasculitides in childhood; the distinguishing features from the adult disease, special risk factors, the prognosis, and the highlights in recent developments in management. Different categories of disorders will be discussed in order of approximate frequency of occurrence.

## Predominantly Small-vessel Vasculitis (Non Granulomatous)

### Henoch-Schönlein purpura

Henoch-Schönlein purpura (HSP) is a small vessel vasculitis that occurs primarily in school-going children. Boys are the common sufferers. Infection with group-A β-hemolytic streptococci is a known trigger factor for the disorder.[2] Other important infectious agents that may precipitate this vasculitis in children include *M.pneumoniae*, parvovirus B-19, hepatitis B virus, and adenovirus. In a series of Brazilian children (n=55) with HSP, infection was identified as the trigger factor in 52.7% of the cases.[3] Some commonly used drugs in childhood may precipitate the occurrence of HSP; these include penicillin, erythromycin, sulphonamides, and anticonvulsants. It is an IgA mediated disorder and abnormality lies in the glycosylation of the hinge region of IgA1 that may be responsible for the pathogenesis.[4]

Onset is usually insidious. Cutaneous lesions start as pink to red maculopapular eruptions that become palpable and purpuric starting initially over the dependent areas like the ankles and legs and progressing upwards to the buttocks and lower back. Pressure points may favor localization of the lesions; occurrence of purpuric lesions on the extensor aspect of the knees of crawling infants may be due to a combined effect of gravity and pressure.[2] Unlike adults, lesions on the face and ears are frequent in infancy, which may be due to a larger head and facial surface area in infants with proportionately higher blood supply.[5] The lesions fade leaving a brownish color that persists for weeks. Other morphological patterns, such as vesicles, erythema multiforme-like lesions, and hemorrhagic bullae, may also be seen, which are rare in children as compared with adults.[6] Infants may develop marked edema of the face, scalp, and extremities.[7]

Childhood disease is usually confined to skin. Arthralgia and/or arthritis, most commonly involving the ankles and knees, may be present. Visceral involvement is rare and if present, renal and gastrointestinal involvements are common. Older children are more at risk of such involvement. Renal disease may precede or follow the cutaneous lesions (unlikely to occur after 3 months but may be seen up to 3 years following skin lesions).[4] Acute abdominal emergency may be the presenting feature of HSP, where the classic cutaneous signs are delayed, sparse, or absent.[8] Gastrointestinal involvements were found to be more common (55.8%) than renal involvement (20.9%) in a recent study of children with HSP.[3] Very rarely, pulmonary hemorrhage may occur with a fatal outcome.[2]

Compared with men, external genitalia is more commonly involved in boys. The clinical features include painless scrotal edema ± purpura, acute testicular pain mimicking torsion[2] or acute onset edema involving the glans penis or prepuce.[9]

The proposed new classification criteria of HSP for children include the following,[1] of which one is a mandatory feature, and at least one of the other four features is compulsory:

- Palpable purpura (mandatory)
- Diffuse abdominal pain
- Biopsy specimen (any involved organ) showing predominant IgA deposition
- Arthritis/arthralgia
- Renal involvement (hematuria and/or proteinuria)

Diagnosis is mostly clinical supported by histopathological features and direct immunofluorescence (DIF) study findings. Imaging procedures (X-ray, ultrasonography, CT scan) are useful to know the type and extent of involvement of the affected organs.

Management protocol includes supportive measures like bed rest, relief of joint pain (paracetamol/codeine) and treatment of any focus of infection; these are sufficient to allow resolution of HSP in most of the cases. Chronic cutaneous involvement in older children may be treated with oral dapsone (25–50 mg/day) (personal experience) or colchicine.[4]

Systemic involvement requires admission to a hospital. Short courses of systemic corticosteroids (1–2 mg/kg) have been used to treat acute abdominal pain and severe scrotal or renal involvement. There is controversy regarding the use of systemic corticosteroids in acute abdominal emergencies as it may mask clinical symptoms and signs. Severe intestinal HSP may respond preferentially to intravenous immunoglobulin and should be considered as first-line therapy.[8] Chronic or recurrent HSP in children has been treated successfully with methotrexate or cyiclosporine.[2]

HSP runs a milder and self-limiting course in children with a good prognosis. The average duration of the illness is 4 to 6 weeks. Single episode disease is more common in infants and younger children. Multiple recurrences may occur in up to 40% of children.[2] The younger the child, the shorter the duration of the disease and incidence of recurrence.

Serious sequelae are uncommon in children. Clinical features with a bad prognosis are pertained to renal involvement; these include reduced glomerular filtration rate (GFR), nephrotic syndrome, and persistence of urinary abnormalities (hematuria and/or proteinuria). Such patients have a higher incidence of hypertension in adult life.[2] Grave complications like cardiac tamponade have been reported in one series of patients.[10] More severe purpuric lesions at the onset is also considered an ominous sign. Mortality in childhood HSP is 1 to 2%, usually related to severe renal or acute gastrointestinal involvement.

### Acute hemorrhagic edema of the skin in infancy

Acute hemorrhagic edema of the skin (Finkelstein disease) is a rare, benign, self-limiting, leucocytoclastic vasculitis of infancy, which has been considered to be a variant of HSP by many authors.

Though factors like preceding infection, immunization, and some drugs have been implicated in etiology, unlike HSP, IgA is not operative here. Activation of a classical complement pathway may be one of the pathogenic mechanisms involved in this disorder.[11]

The age of occurrence varies from 4 months to 2 years. The cutaneous features include ecchymotic purpura involving the face (cheeks, eyelids, and ears) and distal extremities. The lesions start as papules that expand centrifugally to attain a cockade (medallion-like) pattern with scalloped margin and central clearing.[12] Petechial, reticulate, and necrotic skin lesions may be seen. Diffuse tender edema involving the face, eyelids, and ears are seen in 50% of the affected infants. Mild fever and joint pain may be present. Visceral involvement is very rare. There are reports of associated abdominal colic and vomiting, renal involvement, hypocomplementemia, and torsion of the testes. The disease course is 1 to 3 weeks and self resolution is common.

The histopathology of skin lesions reveal intense leukocytoclasia, extravasated RBCs, and fibrinoid necrosis. Some cases may demonstrate perivascular IgA deposits in DIF study (30%), but more frequently IgM, C1q, and fibrinogen are present.[13]

Management is symptomatic. In the presence of infective focus, a course of systemic antibiotics is administered. Monitoring is required only if there are complications.

### Urticarial vasculitis

Urticarial vasculitis (UV) is a cutaneous leucocytoclastic vasculitis presenting with urticarial lesions and may be associated with systemic disorders.

Two clinico-pathological variants, hypocomplementemic UV (HUV) and normocomplementemic UV (NUV) have been described. HUV is associated with significant multi-organ involvement whereas clinical features of NUV are more limited. Hypocomplementemic UV syndrome (HUVS) is a subset of UV with distinct clinical features.

The average age for the onset of UV is the fifth decade, but it has rarely been reported in children as young as 2 years old.[14] Cutaneous lesions are characterized by recurrent, serpiginous wheals associated with a burning and stinging sensation rather than pruritus. The lesions are tender, persist for > 24 hours and heal with hyperpigmentation. There may be associated angioedema.

The most common extracutaneous manifestation includes arthralgia and arthritis. Renal involvement is usually minimal but may progress to end-stage renal failure, even in children. Gastrointestinal, pulmonary, and ocular involvement may occur. Systemic features are more common with HUV and HUVS.

Raised ESR is a consistent laboratory abnormality in patients with UV and help in monitoring the disease. Low complement levels (CH50, C1q, C2-C4) are seen in HUV. Some patients may demonstrate antinuclear antibody. Skin biopsy reveals leucocytoclastic vasculitis.

UV may be associated with SLE or other collagen vascular disorders, infections, cryoglobulinemia, and may follow drug exposure. A careful screening for an underlying cause is essential. Initial treatments include antihistamines with or without antineutrophilic chemotaxis agents like dapsone/colchicine. Systemic involvement requires treatment with corticosteroids and immunomodulators.

### Hypersensitivity vasculitis

Hypersensitivity vasculitis (HV) is a term used to denote leucocytoclastic vasculitis primarily involving the skin, provided other primary and secondary forms of cutaneous vasculitis have been excluded. Sometimes it may be induced by drugs or precipitated by infection. The common causative drugs in children are antibiotics, non steroidal anti-inflammatory drugs (NSAIDs), and anticonvulsants. This form of cutaneous leucocytoclastic vasculitis is uncommon in children compared with patients with HSP. Cutaneous features are similar to HSP. Withdrawal of a responsible drug and symptomatic treatment are the mainstay of treatment.

## Predominantly Medium-sized Vessel Vasculitis

### Polyarteritis nodosa

Polyarteritis nodosa (PAN) is a type of vasculitis affecting predominantly medium-sized blood vessels. The classic PAN is uncommon in children.[12] However, specific subtypes like childhood polyarteritis nodosa and benign cutaneous polyarteritis nodosa are encountered rarely. Systemic manifestations are the predominant clinical features of PAN. The characteristic cutaneous lesions are nodules, ulcers, and livedo reticularis.

Childhood polyarteritis nodosa occurs in children younger than 2 years old. It is a fatal systemic disease mainly involving the heart and kidneys. The affected child presents with fever of unknown origin, malaise, and arthralgia; features of cardiac and renal failure supervene soon thereafter. Cutaneous manifestations include painful nodules that may ulcerate, purpura, and livedo reticularis. Investigations reveal aneurysms of coronary, renal, and cerebral arteries. The prognosis is poor and there is a high rate of mortality.[12]

The proposed classification criteria for childhood PAN are as follows; the histopathologic/angiographic feature is mandatory whereas at least two of the other seven features are compulsory for diagnosis.[1]

- A biopsy showing necrotizing vasculitis involving small or medium-sized arteries/ an angiographic demonstration of an aneurysm/occlusion (mandatory)
- Skin involvement (livedo reticularis, tender subcutaneous nodules, other vasculitic lesions)
- Myalgia/muscle tenderness
- Systemic hypertension relative to childhood normative data
- Mono/polyneuropathy
- Abnormal urinalysis or renal impairment
- Testicular pain/tenderness
- Signs or symptoms suggestive of vasculitic involvement of any other organ (Heart, lungs, central nervous system [CNS], gastrointestinal)

Benign cutaneous polyarteritis nodosa has been defined as a disorder characterized by the presence of cutaneous features but without major organ involvement.[1] Though very rare in childhood, it is the most common variant of the disease seen at this age group.[12] Preceding infection with streptococci or *M. tuberculosis* has been implicated in many cases.[15,16] Other precipitating factors are DPT vaccination, falciparum malaria, and wasp bite.[17]

Skin manifestations include crops of painful, erythematous, subcutaneous nodules present mostly on the lower legs, frequent ulcerations, urticaria, and livedo reticularis. The nodules are distributed all over, including around the malleoli and are in different stages of evolution.[18] Following healing, a livedoid/violaceous, retiform pigmentation persists for years. There is a higher incidence of peripheral gangrene in children. Limited extra-cutaneous manifestations include myalgia, arthralgia, non-erosive arthritis and peripheral neuropathy. The disorder runs a chronic course with relapses and remissions. All children should be followed-up closely for evolution of systemic symptoms.[4]

Leukocytosis, thrombocytosis, high ESR, and raised C-reactive protein are usual in cases of childhood PAN, of which ESR is the most commonly altered parameter. In cutaneous PAN, acute phase reactants are often normal.[4] Anti-streptolysin O (ASLO) titer may be raised and some authors recommend estimation of ASLO titer in all patients with cutaneous PAN.[15] Antineutrophil cytoplasmic antibody (ANCA) is negative in cutaneous PAN which is a distinguishing feature from childhood PAN where perinuclear ANCA (p-ANCA) is positive. Biopsy from skin lesions/involved viscera reveals necrotizing arteritis. Inflammatory changes in a single artery in the deep dermis or subcutaneous fat and focal panniculitis around the involved artery are the characteristic cutaneous histopathology.[18] Conventional or 3-D magnetic resonance angiography is helpful in detecting aneurysms.[4]

In the presence of fever and coronary aneurysm, childhood PAN may be confused with Kawasaki disease; the fatal outcome in the former is the distinguishing feature.

Erythema nodosum presents with nodular lesions on pretibial areas, whereas the subcutaneous nodules of PAN are distributed all over.[4] Histopathologically, other nodular disorders of the leg show more diffuse panniculitis compared with nodules of cutaneous PAN.[18]

Patients with milder, limited cutaneous disease may be managed conservatively with NSAIDs or salicylates.[19] A course of antibiotics is administered if there is evidence of infection.[16,18] Systemic corticosteroids are the mainstay of treatment in non responders to above therapeutic modalities and moderate to severe disease. In the presence of renal involvement in case of childhood PAN, a combination of systemic corticosteroids and cyclophosphamide or azathioprine is effective.[4,20] Intravenous immunoglobulin, methotrexate (weekly dosage), and plasma exchange are the other therapeutic modalities that have been tried for childhood PAN. Recently, infliximab has been used successfully in treating patients with childhood cutaneous PAN.[21] Penicillin prophylaxis may be considered in children with cutaneous PAN to prevent recurrence.[4]

### Kawasaki disease

Kawasaki disease (KD) is a unique self-limiting vasculitis seen in childhood, the sequelae of which may continue until adulthood. Most of the reported cases are from Japan; Japanese children are supposed to have high genetic susceptibility for KD, whereas cases of KD are low in Europe and North America.[22] Recently, clusters of cases have been reported from India.[22] Approximately 85% of the children are younger than 5 years; patients older than 3 years and younger than 3 months old are encountered very rarely.[23] Boys are more commonly affected. Clinical presentation may not be uniform in all cases and an incomplete KD may be seen, especially in younger infants.

The etiopathogenesis of KD is unclear. The fact that KD simulates many infective exanthema of childhood and there are reports of preceding infection in some, lead to a consideration of infectious etiology. It is probably a multifactorial disease occurring in genetically predisposed children, precipitated by some bacterial super antigens that results in host immune activation. The toll-like receptor-4 signal pathway, which activates nuclear transcription factor kappa β and induces excessive production of proinflammatory mediators, are found to be significantly activated during acute KD.[24]

The child presents with abrupt-onset and high fever (>39.0°C) without any prodrome, which shows poor response to antipyretics and remains undiagnosed for prolonged periods.[12] There is extreme irritability. A non-specific, polymorphous skin rash (diffuse macular/papular with a sandpaper-like feeling/urticarial/pustular/erythema multiforme-like) may appear at some point during the illness, usually within the initial 5 days. Skin lesions characteristically tend to localize in the flexures, especially in the groin, which may evolve into a desquamating perineal eruption with a bright background erythema.

Diffuse palmo-plantar erythema appears within 5 days of the onset of illness with brawny, non-pitting edema of the hands and feet. Digital tip and periungual fissuring and desquamation start after 10-15 days and may spread to involve the palms, soles, and wrists.

Mucosal features include a non exudative conjunctivitis primarily involving the bulbar conjunctiva (that appear within 2 to 4 days), dry, red, fissured/crusted lips, erythema of the oral mucosa, and red strawberry tongue. Lip and oral mucosal erythema may persist for a few weeks even after subsidence of other features. Characteristic bilateral conjunctivitis and red, fissured lips give the patients a typical look that should raise the suspicion of KD in a febrile child.

Peripheral ischemia and gangrene of the digits may occur rarely. Following recovery, in later stages, nails may show Beau's lines.

A feature noted in younger infants with KD is erythema and induration at the bacillus Calmette-Guérin (BCG) inoculation site during the acute stage of the disease.[25] Some authors consider it to be a specific sign of KD and recommend it as an early diagnostic tool in an incomplete form of the disease.[25] This feature is particularly helpful in diagnosing KD in developing countries like India, where BCG vaccination is routine.[25]

Cervical lymphadenopathy is observed in 50-75% of the patients[12] with a size of 1.5-5 cm. Different internal organs are variably involved [Table 2]. Cardiac and coronary arterial involvement during the acute stage of the disease as well as thereafter is one of the important determinants of the prognosis of the disease.

**Table 2:** Systemic involvement in Kawasaki disease[12]

System involvement	Clinical features	Laboratory features
Central nervous system involvement	Irritability Lethargy Features of meningeal irritation Cerebral infarction Cranial nerve palsy	CSF mononuclear pleocytosis
Cardio-vascular involvement	Early stage	
	Pericarditis and effusion Myocarditis (tachycardia and gallop rhythm) Congestive heart failure Arrhythmia	2-D echocardiography Coronary angiography
	Late stage	
	Mitral valvular regurgitation Coronary aneurysm	
Hepatobiliary involvement	Hepatitis and jaundice Hepatomegaly Hydrops of gallbladder	↑Serum bilirubin and transaminases ↓Serum albumin
Gastrointestinal involvement	Diarrhea	
Hematological involvement	Anemia	Normochromic, normocytic peripheral smear Leukocytosis Thrombocytosis / thrombocytopenia ↑ESR
Uro-genital involvement	Urethritis	Sterile pyuria Occasional mild proteinuria
Musculo-skeletal involvement	Arthralgia and arthritis	
Eye and ear involvement	Anterior uveitis Sensorineural deafness	

KD has to be differentiated from other febrile exanthematous diseases of childhood. These include scarlet fever, viral exanthemas, maculopapular drug rash, and early Stevens Johnson syndrome. Recurrent toxin-mediated perineal erythema, an entity described in adults, has recently been reported in a series of pediatric patients.[26] It is a febrile illness with a sudden appearance of salmon-colored perineal erythema and rapid desquamation simulating KD. The conjunctivitis of KD is distinct by the presence of a peri-limbal clear halo, which is absent in infective or other inflammatory disorders of eye.[12]

Diagnosis of KD is clinical. The proposed new classification criteria for KD are as follows,[1] of which one is mandatory and four of the other five criteria are required for diagnosis:

- Fever for a minimum period of 5 days (mandatory criteria)
- Desquamation in peripheral extremities and perineum
- Polymorphous exanthema
- Bilateral conjunctival injection
- Changes in the lips, oral cavity or injection of the oral and pharyngeal mucosa
- Cervical lymphadenopathy

Most of the cases pose a diagnostic challenge, more so the incomplete forms. Considering KD as a differential diagnosis in all febrile, irritable children with mucocutaneous changes reduces the possibility of missing the diagnosis.

Starting treatment early (within 10 days) with anti- inflammatory agents is of immense importance to prevent sequelae. The main therapeutic agents are aspirin and intravenous immunoglobulin (IVIG). The current practice is to administer a single dose of IVIG (2 g/kg). Simultaneous treatment with aspirin (80-100 mg/kg/day), until the fever subsides, is recommended to achieve its maximum anti- inflammatory effect.[12] There is rapid remission of the fever and conjunctivitis with this combined treatment. Thereafter low-dose aspirin (3-5 mg/kg/day) is continued for its anti-platelet effect until normalization of laboratory parameters.[12] Some patients (15-20%) may be non responsive to IVIG therapy; intravenous steroid pulse (methyl prednisolone 30 mg/kg over 2 hours) followed by IVIG (2 g/kg) over 24 hours has been used in these patients with success.[27] Infliximab is helpful in treatment of refractory cases and also in the presence of coronary aneurysm.[28]

Patients with KD are left with cardiovascular sequelae. Infants with a longer duration of fever and incomplete clinical features are at a higher risk of developing coronary artery aneurysm.[29] Some patients (5-10%) may continue to develop coronary aneurysm even following adequate treatment;[12] hence long-term screening of these patients with echocardiography is required. Sudden death may occur within 3 to 8 weeks of the onset of the illness.

## Predominantly Small-vessel Vasculitis (Granulomatous)

This group of vasculitides occur mainly in adults and children are affected very rarely. The prototype disorder is Wegener's granulomatosis (WG), which is a necrotizing granulomatous vasculitis affecting mainly the upper and lower respiratory tracts, ears, and kidney; other organ involvement are seen to some extent. Adult and childhood/adolescent WG show an identical clinical spectrum except for a higher central nervous system (CNS) involvement in adults.

In a review of childhood WG, skin lesions were recorded in 9% of children at the onset of the disease and in 52% of the children, skin was affected during the course of the disease.[30] Skin lesions are non specific, including palpable purpura, necrotic papules and nodules, vesicles/pustules and ulcers; these are indistinguishable from the skin lesions of Churg-Strauss syndrome, another disorder in this group. Large ulcers simulating pyoderma gangrenosum but lacking the rolled-out margin are pathognomonic of WG.[31] Necrotizing vasculitis involving the anus and perianal skin extending to the rectum may be a feature.[17]

Gingival hyperplasia may occur in the form of exuberant, spongy tissue, studded with petechiae, especially involving the interdental papillae (strawberry gingival hyperplasia). Ulceration may occur in palate. Nasal deformity (saddle nose, septal perforation) is more common in children and adolescents compared with adults. Proptosis may result from granulomatous pseudotumor involving the orbit.[17]

A protracted superficial variant of WG exists, wherein necrotic ulcers are localized to the skin (face, fingers, and toes) and mucous membrane and the disease runs a milder course with delayed development of systemic features. This variant has also been reported in children.[32]

Skin constitutes an accessible, easy site for biopsy in patients with WG with lesions. Histopathology reveals characteristic necrotizing granulomatous vasculitis, suggestive but not confirmative of WG. Cytoplasmic ANCA (c-ANCA) is positive in 80% of the cases. Systemic glucocorticoid and immunosuppressives (cyclophosphamide, methotrexate, and azathioprine) are the mainstays of therapy.

## Predominantly Large Vessel Vasculitis

Takayasu's arteritis is a granulomatous vasculitis involving the aorta and its branches. It is an adult onset disorder and rare in children. Twenty percent of patients have disease onset before the age of 19.[4] Thoracic and abdominal aorta are the commonly affected segments in this early-onset disease. Patients present with systemic features like fever, night-sweats, arthralgia, anorexia, and weight loss. Hypertension or congestive heart failure may be the presenting features. Cutaneous features are subtle and include erythema nodosum-like lesions.

## Other Vasculitides

### Behçet's disease

Behçet's disease (BD) is a multisystem vasculitic disorder affecting the arteries and venules along with thrombotic tendency.[4] Only 1–2% of the affected patients with BD are in the pediatric age group and family members (parents and siblings) may be affected.[4,33] Usually, this affects older children, rarely infants.[33]

The pathogenesis of BD is unknown. Genetic susceptibility (more frequent in HLA-B5 individuals) may be present and trigger factors like infection (herpes simplex virus, Streptococci) may precipitate the disease.

Major clinical features include recurrent oro-genital aphthosis and uveitis, but multi-organ involvement is present [Table 3]. In an international collaborative study on childhood BD, painful oral ulcers (major/minor/herpetiform) were present in almost all cases (96%) involving the lips, gingiva, buccal mucosa, tongue, tonsils, and palate.[33] Genital aphthae usually follow oral lesions (70%) and involves the scrotum, penis, and vulva and heal with scarring. Perianal ulceration may be present in some cases, which may be considered as a specific feature of childhood BD.[33]

**Table 3:** Organ involvement and related manifestations in Behçet's disease[17]

System involvement	Clinical features
Central and peripheral nervous system	Headache, dementia, psychiatric abnormality
	Meningoencephalitis
	Cerebral venous thrombosis
	Cerebellar signs
	Benign intracranial hypertension
	Hyperaesthesia, peripheral nerve palsy
Gastrointestinal involvement	Aphthous ulcers throughout GI mucosa
	Nausea, vomiting, diarrhea, weight loss (may mimic inflammatory bowel disease)
Cardiovascular involvement	Thrombophlebitis
	Aortic aneurysm
	Pericarditis
	Myocarditis
Pulmonary involvement	Hemoptysis due to pulmonary arterial involvement
Renal and genito-urinary involvement	Hematuria
	Proteinuria / nephrotic syndrome
	Urethritis
	Orchitis and epididymitis
Hematological involvement	Thrombocytopenia
	Neutropenia

Skin lesions are non specific and are of common occurrence (92%) in children.[33] These include erythema nodosum, folliculitis, pustular eruptions, and palpable purpura. Necrotic folliculitis is more common in boys.[33] There are reports of skin lesions simulating other dermatoses such as erythema multiforme, pyoderma gangrenosum, and Sweet's syndrome.[4] There is ethnic variation of clinical features and cutaneous manifestations are more common among Turkish children with BD.[4,33]

The following are the international classification criteria for BD, of which the mandatory criterion and two of the other criteria must be present:[17]

- Recurrent (≥3 episodes per year) oral aphthous ulcers (mandatory)
- Recurrent genital aphthae
- Ophthalmic lesions: Anterior/posterior uveitis, cells in vitreous humour on slit lamp examination, retinal vasculitis
- Skin lesions: Erythema nodosum, pseudofolliculitis, papulopustular acneiform nodules
- Positive pathergy test

The clinical spectrum of adult and childhood BD is similar except that ophthalmic involvement is rarer in children. Neurological involvement and thrombophlebitis are also rare in children. Arthritis involving the lower limbs (knee or ankle) may be the presenting feature. Acute myocardial infarction has been reported in childhood BD, which is a very rare complication.[34]

A single episode of oral ulceration can be managed with topical corticosteroids and antiseptic mouthwash. Recurrent episodes of orogenital ulcerations require systemic therapy with either colchicine, corticosteroids, or thalidomide. Skin lesions can be managed with topical corticosteroids and colchicine. Other drugs found to be effective in children are azathioprine, cyclosporine, and mycophenolate mofetil. In the presence of CNS involvement, systemic corticosteroids in combination with cyclophosphamide or chlorambucil are used.

The prognosis of childhood BD is variable. Females have a relatively benign course of the disease compared with boys.[33] Features with a poor outcome are encephalopathy, severe uveitis, and multiple venous thromboses; the latter two being more common in boys.[33] Mortality was found to be 3% in a series of patients with childhood BD.[33]

Cutaneous vasculitis may rarely be seen in children with internal malignancy as a paraneoplastic syndrome.[35] *Mycoplasma pneumoniae* pneumonia is common in childhood and may be associated with cutaneous vasculitis.[36] ANCA-associated vasculitis has been reported in a child receiving anti-thyroid medication.[37]

A majority of the children with cutaneous vasculitis have a self-limiting disorder. However, systemic involvement is a common accompaniment and early recognition of these features is crucial in the management. The vasculitides with predominant systemic manifestations may present initially to pediatric specialists, but dermatological consultation is often sought in due course of the disease when cutaneous features appear. The rarity of vasculitides in childhood period allows the clinicians to ignore these as differential diagnosis in many situations with a diagnostic dilemma. However, a searching eye and a careful mind may pick up some of these rare cases.
